# Combination of Synergic Enzymes and Ultrasounds as an Effective Pretreatment Process to Break Microalgal Cell Wall and Enhance Algal Oil Extraction

**DOI:** 10.3390/foods10081928

**Published:** 2021-08-19

**Authors:** Cristina Blanco-Llamero, Paz García-García, Francisco Javier Señoráns

**Affiliations:** Healthy-Lipids Group, Departmental Section of Food Sciences, Faculty of Sciences, Universidad Autónoma de Madrid, 28049 Madrid, Spain; cristina.blanco@uam.es (C.B.-L.); mariap.garcia@uam.es (P.G.-G.)

**Keywords:** microalgae, enzymatic pretreatment, Viscozyme^®^, Alcalase^®^, Celluclast^®^, *Nannochloropsis gaditana*, polar lipids, sonication

## Abstract

Microalgal biomass is a sustainable source of bioactive lipids with omega-3 fatty acids. The efficient extraction of neutral and polar lipids from microalgae requires alternative extraction methods, frequently combined with biomass pretreatment. In this work, a combined ultrasound and enzymatic process using commercial enzymes Viscozyme, Celluclast, and Alcalase was optimized as a pretreatment method for *Nannochloropsis gaditana*, where the Folch method was used for lipid extraction. Significant differences were observed among the used enzymatic pretreatments, combined with ultrasound bath or probe-type sonication. To further optimize this method, ranges of temperatures (35, 45, and 55 °C) and pH (4, 5, and 8) were tested, and enzymes were combined at the best conditions. Subsequently, simultaneous use of three hydrolytic enzymes rendered oil yields of nearly 29%, showing a synergic effect. To compare enzymatic pretreatments, neutral and polar lipids distribution of *Nannochloropsis* was determined by HPLC–ELSD. The highest polar lipids content was achieved employing ultrasound-assisted enzymatic pretreatment (55 °C and 6 h), whereas the highest glycolipid (44.54%) and PE (2.91%) contents were achieved using Viscozyme versus other enzymes. The method was applied to other microalgae showing the potential of the optimized process as a practical alternative to produce valuable lipids for nutraceutical applications.

## 1. Introduction

In recent decades, microalgae have been an interesting biomass source due to their potential to rapidly accumulate important amounts of added value components that vary among the different species. Moreover, their high areal productivity that does not require arable lands enables their use as a promising and environmental-friendly source of bioactive compounds. Nevertheless, the large scale and cost-effective manufacture of these compounds are currently quite challenging in terms of both production and purification. An integrated bioprocessing approach using microalgae thus needs to consider both the upstream production of microalgae and the downstream harvesting and extraction. New cell disruption procedures must be developed to overcome problems associated with traditional expensive pretreatment methods. Considering all these facts, it is important to highlight that each process must be designed on each microalgae species due to their uniqueness, which makes the entire process still challenging.

Among different microalgae that are harvested worldwide, *Nannochloropsis gaditana* is described to produce a wide range of polar lipids, including omega-3 polyunsaturated fatty acids (PUFAs) and carotenoids such as lutein, which have widely recognized benefits related to human health. Indeed, PUFA produced by microalgae exhibit antioxidant, antibacterial, antiviral, and detoxifying capacities; prevents hypercholesterolemia; improves brain function; and is proved to have good immune-stimulatory effects [1,2]. *Nannochloropsis gaditana* has been described to produce high amounts of lipids. Specifically, it has an important content of triglycerides (TAG) and polar lipids, such as phospholipids (PLs) and glycolipids (GL). Polar lipids are important structural and functional components of cell membrane where microalgae have the capacity to accumulate high levels of eicosapentaenoic acid (EPA) [3,4,5].

Additionally, in many of the lipid sources and in the commercialized formulas, EPA and docosahexaenoic acid (DHA) are bounded to TAG or ethyl esters (EE), which affects their nutritional properties making them less bioavailable for the human body, whereas one of the main characteristics of lipid producer microalgae is the presence of these fatty acids bound to PLs and GL, which are more adequate nutritional forms regarding on bioavailability of the compounds and its bioactivity. Thus, fatty acids can be directly extracted from microalgae in these polar forms (PLs and GL) and commercialized without any extra industrial process, making microalgae a promising nutritional source of bioactive polar lipids [5,6,7,8,9].

However, one of the most critical points in the extraction of bioactive ingredients from microalgae is the selection of an appropriate extraction technique due to the presence of a dense and firm cell wall. The rigid cell wall of *N. gaditana* contains proteins and other biopolymers such as cellulose or pectin, among others [10]. For that reason, it must be properly disrupted to efficiently recover intracellular bioactive compounds [11].

Nowadays, the vegetable oil extraction industry uses large volumes of hexane and long extraction times that cause environmental problems due to direct losses of organic solvents into the atmosphere; hence, greener alternatives are needed. In recent years, there have been important advances in the development, optimization, and applications of green extraction techniques in the food industry to reduce the environmental impact by waste minimization, decrease energy consumption and processing time, and avoid health hazards by substituting toxic and unsafe solvents and developing green pretreatment methods that facilitate posterior oil extraction [1,3,11].

Ultrasound-assisted extraction (UAE) has been demonstrated to be a promising alternative to conventional extraction in order to reduce energy consumption and increased the extraction yield for lipids and carotenoids [12,13]. Cavitation is considered the fundamental mechanism for UAE, where micro-bubbles form and collapse near the cells, creating cellular disruption. Furthermore, UAE has the benefits of higher efficiency, reduced amount of solvent and extraction time, moderate cost, and simple handling [14,15]. Additionally, ultrasonication can usually be carried out at a low temperature, which reduces potential thermal damage to bioactive ingredients or the loss of volatile components during extraction or pretreatment. Enzymatic degradation of algal cell walls prior to lipid extraction has the potential to facilitate both lipid extraction and post-extraction use of the algal biomass. Weakened cell walls could reduce solvent and energy inputs needed for lipid extraction by either liberating cell wall mono and polysaccharides or improving accessibility of cell wall polymers to microorganisms, enzymes, or reagents [16,17,18,19]. Thus, the use of enzymes facilitates the hydrolysis of microalgae cell walls, and if enzymes are combined with other physical disruption methods, fast extraction and the highest extraction yields could be obtained.

Previous studies of the research group demonstrated that it was possible to enrich and fractionate neutral and polar lipids using enzymatic pretreatment and pressurized liquid extraction (PLE) from microalgae [1,3,12]. In the present work, the combination of different commercial enzymes, including a wide range of carbohydrases (Viscozyme^®^ and Celluclast^®^) and protease (Alcalase^®^), were tested in combination with other methods to enhance and optimize polar lipid recovery of *N. gaditana* dry biomass.

## 2. Materials and Methods

### 2.1. Materials

*Nannochloropsis gaditana* dry biomass was provided by Algaenergy S.A. (Alcobendas, Spain).

Chloroform and isopropyl alcohol were purchased from Scharlab S.L. (Sentmenat, Spain). Methanol was purchased from Lab Scan Analytical Sciences (Gliwice, Poland). Hexane and HPLC-grade solvents (2,2,4-trimethyl pentane, methyl tert-butyl ether (MTBE)) were purchased from Macron Fine Chemicals (Gliwice, Poland). Absolute ethanol (PRS grade), sodium hydrogen carbonate, and potassium hydroxide were purchased from Panreac Química S.A (Barcelona, Spain). The water used was Milli-Q grade (Millipore Sigma, Burlington, MA, USA). Viscozyme^®^ from *Aspergillus aculeatus* containing a wide range of carbohydrases, including arabinase, cellulase, beta-glucanase, hemicellulase, and xylanase, and Celluclast^®^ containing cellulase from *Trichoderma reesei* and Alcalase^®^ were kindly donated by Novozymes (Bagsvaerd, Denmark). Glyceryl trilinoleate, dioleoylglycerol (mixture of 1,3- and 1,2-isomers), 1-oleoyl-rac-glycerol, oleic acid, and ethyl linoleate used as HPLC standards was purchased from Sigma-Aldrich (St. Louis, MO, USA). All other reagents and solvents used were of analytical or HPLC grade.

### 2.2. Bradford Method for Protein Quantification

The protein concentration was determined by the method of Bradford [20]. The samples were diluted (1/2, 1/5, 1/10, 1/20) to obtain different enzymatic solutions. To perform the measurements, 20 μL of the sample was added to 1 mL of Bradford’s solution and allowed to react for 30 min. The absorbance was measured at 595 nm on a model UV-Vis UV-1280 spectrophotometer. The absorbance range of the samples must be between 0.1 and 1 for measurements to be reliable. Different concentrations were obtained from a known standard curve for bovine serum albumin (BSA). Protein determinations were performed at least in duplicate in all cases.

### 2.3. Enzymatic Pretreatment under Different Conditions

The protocol of the enzymatic pretreatment of *Nannochloropsis gaditana* biomass was adapted from the procedure described by Zuorro et al. [21]. One gram of dry microalgal biomass was resuspended in 10 mL of sodium citrate buffer 0.1M pH 5.0 containing 46 mg of different enzymes (Viscozyme^®^, Alcalase^®^, and Celluclast^®^) per gram of biomass. In addition, 1 g of biomass was resuspended in a 10 mL buffer without enzymes (blank solution). The solutions were incubated during different times under different conditions (thermal incubation, ultrasound bath incubation, and sonication).

Thermal incubation was developed at 55 °C using a Heidolph Incubator Unimax 1010 (Schwabach, Germany) with agitation at 200 rpm, and samples were obtained at different times (6, 12, and 24 h).

Ultrasound (US)-assisted enzymatic pretreatment was carried out with a US bath Elmasonic S 40H Elma brand (Singen, Germany) with automatic control of time (2, 4, and 6 h) and temperature (55 °C), US frequency of 37 kHz and 140 watts bath power.

Sonication of the enzymatic pretreatment was accomplished using a Brandson 450 Digital Sonifier w/ Probe for 15 and 30 min in an ice bath with intervals of 7 s ON and 12 s OFF with 400 watts of power. After different incubation times, the flask content was centrifuged at 3000 rpm for 10 min, the supernatant was discarded, and pellet biomass was kept at 4 °C for its extraction and characterization. Experiments were performed at least in triplicate in all cases.

### 2.4. Optimization of Ultrasound-Assisted Enzymatic Pretreatment

Considering the optimum pH and temperature range of each commercial enzyme, different conditions were studied. One gram of dry microalgal biomass was resuspended in 10 mL of sodium citrate buffer 0.1M at different pH (4.0, 5.0, and 8.0) containing 46 mg of commercial enzymes and incubated at different temperatures (35, 45, and 55 °C) in a US bath for 6 h. In addition, 1 g of biomass was resuspended in a 10 mL buffer without enzymes (blank solution). The flask content was centrifuged at 3000 rpm for 10 min, the supernatant was discarded, and pellet biomass was kept at 4 °C for its extraction and characterization. Experiments were performed at least in triplicate in all cases.

### 2.5. Combination of Enzymes under Optimized Pretreatment Conditions

Enzymatic combo solution containing 46 mg of commercial enzymes (Viscozyme and Celluclast, or Viscozyme, Celluclast, and Alcalase) was incubated under optimal conditions (pH 5.0 and 55 °C) in US thermal bath. The flask content was centrifuged at 3000 rpm for 10 min, the supernatant was discarded, and pellet biomass was kept at 4 °C for its extraction and characterization. Experiments were performed at least in triplicate in all cases.

### 2.6. Lipid Extraction of Microalgal Biomass

Lipid extractions from microalgal biomass from *Nannochloropsis gaditana* were carried out using the conventional standard Folch method [22]. The experiments were done at least in duplicate in all cases.

The Folch extraction method was performed following the original procedure described by Folch et al. [22]. Microalgal biomass (1 g) was extracted with 20 mL of chloroform:methanol (2:1) vortexing for 2 min. The mixture was centrifuged at 3000 rpm for 10 min, and the organic layer was collected. The collected organic layers were purified washing with water and centrifuged at 3000 rpm for 10 min. The purification process was carried out three times on the same organic layer. Finally, the chloroform layer containing the extracted lipids was collected and evaporated.

The samples were evaporated in a rotary evaporator (Heidolph Hei-Vap Value HB/G3, Germany) under reduced pressure at 40 °C and dried under a nitrogen stream until constant weight. The lipid content was determined gravimetrically and calculated as a weight percentage of dry biomass. Lipid extracts obtained were stored in dark vessels with a nitrogen atmosphere at 4 °C until their analysis.

### 2.7. HPLC–ELSD Analysis

HPLC with Evaporative Light Scattering Detector (HPLC–ELSD) analyses was performed using an Agilent 1260 Infinity HPLC equipped with an Agilent 385 (Palo Alto, CA, USA) ELSD instrument. The chromatographic separation of the different species of lipids (neutral and polar lipids) was performed with a silica normal-phase ACE (250 mm × 4.6 mm i.d. 0.5 µm) column maintained at 30 °C using a ternary gradient as follows: 0–3 min, 95% B and 5% C, 50% B and 50% C; at t = 3 min, 2% A, 48% B, and 50% C; at t = 9 min, 60% A and 5% B; and 35% C at t = 17 min, 75% A and 5% B and 20% C at t = 21 min, 50% B and 50% C at t = 31 min, and 95% B and 5% C at 33 min. Eluent A consisted of methanol, eluent B consisted of 2,2,4-trimethylpentane, and eluent C consisted of methyl tert-butyl ether. The flow rate was variable (1.0 or 2.0 mL/min) and programed. The optimal signal and resolution were achieved with the following ELSD conditions: evaporator temperature = 30 °C; nebulizer temperature = 30 °C; evaporator gas N2 = 1.6 SLM.

Lipid species were identified using commercial standards for neutral lipids like TAG, DAG, MAG, GL, and FFA. Results were expressed as the individual relative percentage of each lipid species present in the sample (normalized areas). HPLC–ELSD analyses were performed at least in duplicate in all cases.

### 2.8. Statistical Analysis

The results were expressed as the mean of the experiments and its standard deviation. Statistical analysis was performed in the SISA (Simple Interactive Statistical Analysis) online software available at http://www.quantitativeskills.com/sisa/statistics/t-test.htm (accessed on 20 May 2021). The data were subjected to a t-test to examine whether the two groups mean differ from one another. To test if there is an overall statistically significant difference between three or more means, the data were subjected to a one-way analysis of variance (ANOVA) using the F test for discrimination between means (*p* < 0.05).

## 3. Results and Discussion

### 3.1. Enzymatic Pretreatment in Incubator

**Figure 1:**
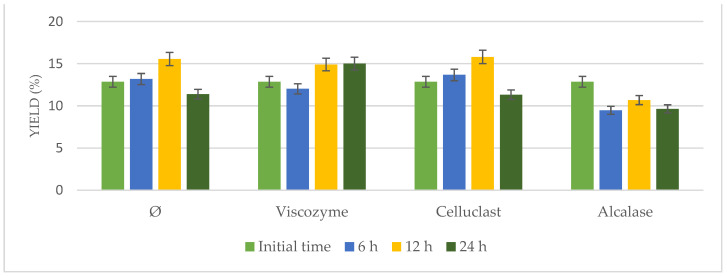
Oil yield of pretreated *N.gaditana* biomass after enzymatic incubation at 55 °C at different times (6, 12, and 24 h) compared to not pretreated biomass (Ø) under the same conditions.

Enzymatic pretreatment was applied to *N. gaditana*, adding Viscozyme, Alcalase, or Celluclast under continuous stirring in an incubator for different times (6, 12, and 24 h). Results in terms of yield extraction were compared to *N. gaditana* incubated under the same conditions in the absence of enzymes (Figure 1).

Results hardly varied from 12.85% to 16.32%. Increased yields were observed at longer times (12 h), either using enzymatic pretreatment or not, achieving similar results (*p* > 0.05). Taking into account the yield of the initial time and that achieved by the biomass incubated without enzymes, it was possible to confirm that the observed effects were attributed to time and temperature under stirring but not to enzymatic addition. These results could be due to the rigid cell wall of the microalgae and the difficulties of the enzymes to get into the cells.

Researchers have reported in literature many results of enzymatic degradation; however, the enzymatic process did not achieve very high cell disruption degrees. Most of the reported studies have used enzymes separately and have also had limited lipid extraction. Thus, for improving the enzymatic disruption of algal cells, a synergy of different kinds of processes and enzymes might be applied [23].

### 3.2. Enzymatic Pretreatment Coupled with Ultrasound-Assisted Pretreatment

Enzymatic pretreatment in combination with physical pretreatments such as ultrasonication was tested to enhance the enzyme’s action on the selected microalgae.

Ultrasonication has been intensively used for microalgal cell disruption [23,24]. However, ultrasonication on its own had not given very high disruption efficiency. In this project, the combination of ultrasonication coupled with enzymatic pretreatment as a synergetic method was studied. This method was compared to ultrasonication on its own. Several variables such as the enzyme used and the time of the process were studied to achieve higher disruption rates, decrease the time of the process, and increase the extracted *N.gaditana* oil yield.

**Figure 2:**
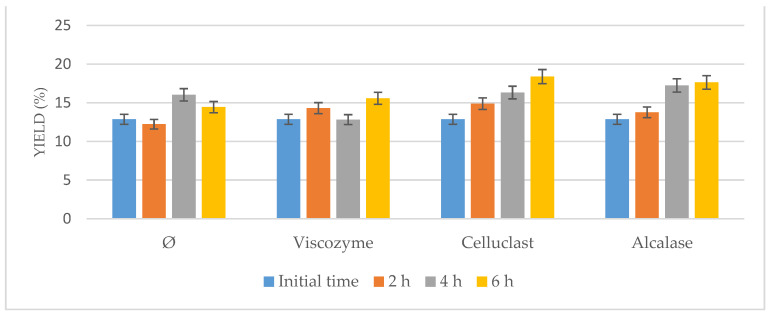
Ultrasound-assisted enzymatic pretreatment at 55 °C, pH 5.0 and different times (2, 4, and 6 h) compared to not pretreated biomass (Ø) under the same conditions.

As it can be seen in Figure 2, the oil yield increased using only the US bath, suggesting the US-assisted pretreatment itself could be a useful method to enhance lipid recovery from *Nannochloropsis gaditana* as had been described [25]. However, higher results were obtained when enzymes were added to the process either using one or another (*p* < 0.05), improving the results that the US achieved when they were tested on their own.

Moreover, an increase in time of the process up to 6 h implied a higher oil yield for all the conditions tested because it favored the action of the enzymes in combination with the US. No further improvement was observed after 6 h (results not shown), achieving similar results when 8 h and 12 h was used instead. Consequently, the optimal time of the process was determined as 6 h.

On the other hand, comparing amongst the different enzymes, it could be seen how at the same concentration, Celluclast achieved the highest results closely followed by Alcalase and Viscozyme, which suggests that most of the microalgae cell wall is composed of cellulose, which is in agreement with previous works on cell wall composition for *Nannochloropsis* [16,25].

Thus, depending on the used enzymes and the different conditions in which enzymatic reaction was carried out, different temperatures and pressures caused by the cavitation phenomenon were achieved during the US action, and consequently, different mass transfer rates and degrees of cell wall disruption of the biomass were produced, resulting in different oil contents released into the medium [26].

### 3.3. High-Intensity Sonication Combined with Enzymatic Pretreatment

**Figure 3:**
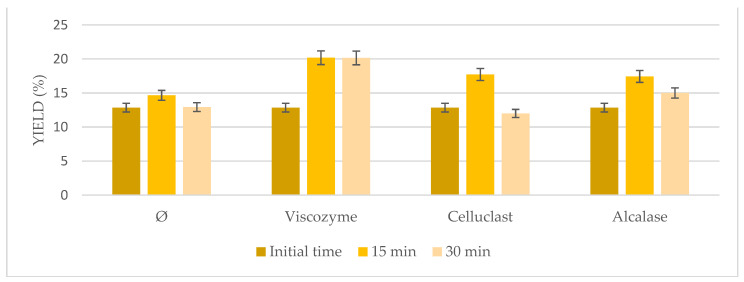
Oil yield obtained from high-intensity sonication at 4 °C combined with enzymatic pretreatment using *Nannochloropsis gaditana* compared to not pretreated biomass (Ø) under the same conditions.

Based on the interesting results with the US bath, probe-type sonication as a more energetic method was tested in combination with enzymatic pretreatment at shorter times (Figure 3). In general, even higher results than the ones achieved using the US bath were obtained (*p* < 0.05) when both pretreatments were combined, sonication probe and enzymatic pretreatment, ranging from 12.85% to 20.26%. Indeed, comparable results to the US bath were obtained at only 15 min instead of 6 h, reducing time and the high energy cost of the entire process, resulting in a more energy effective process. Not very interesting results were obtained when sonication was used on its own, achieving similar results to the initial time. However, the highest results were obtained when Viscozyme was used at 15 min (20.26%), followed by Celluclast (18.14%) and Alcalase (17.51%). On the other hand, increasing time to 30 min seemed to have negative effects on the microalgae extraction, which may be due to the very aggressive conditions used that may damage the microalgae biomass.

### 3.4. Optimization of the Ultrasound Enzymatic Pretreatment

Based on the described results, an optimization of the US-assisted enzymatic pretreatment was tried regarding oil yield, pH, and temperature of the process.

**Figure 4:**
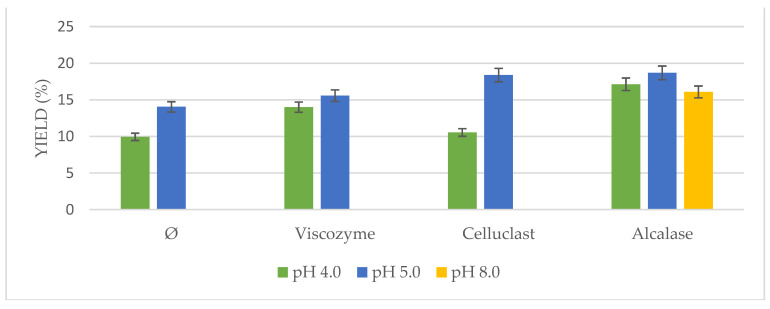
*Nannochloropsis gaditana* oil yield obtained by ultrasound-assisted enzymatic pretreatment at different pH (4, 5, and 8).

On the one hand, different conditions at the optimum pH range for each enzyme were studied (Figure 4). Significant differences in oil yields were found at different pH solutions for all enzymes. Results ranged from 12.85% to 21.86%. In the case of the non-pretreated biomass, a pH of 5.0 had a higher influence on the posterior extraction, as it occurred as well using enzymes addition. Optimum pH for Viscozyme and Celluclast ranged from 4.0 to 5.0 in the literature, whereas optimum pH for Alcalase was described to be 8.0. However, it is interesting to point out how at pH 5.0, Alcalase achieved similar yields to the ones observed at pH 8.0 in the present study, on the contrary of the previous works with this enzyme [27,28].

**Figure 5:**
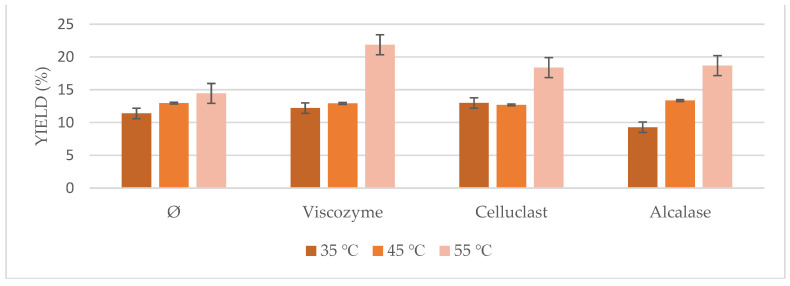
*Nannochloropsis gaditana* oil yields obtained by ultrasound-assisted enzymatic pretreatment at different temperatures (35, 45, and 55 °C).

Based on these results, the optimum pH found for each enzyme was used to test the temperature of the process (Figure 5). Conditions of 55 °C seemed to be the optimum for all of the cases, either using pretreated biomass or not, which is in agreement with most of the previous works on enzymatic pretreatment using Viscozyme, Celluclast, and Alcalase, where their optimum temperature ranged from 35 to 55 °C, achieving its maximum at 55 °C [27,29,30,31,32].

Thus, the optimal conditions for the synergic effect of enzymatic pretreatment and US incubation were incubation for 6 h at pH 5.0 and 55 °C for all enzymes studied (Viscozyme, Celluclast, and Alcalase).

### 3.5. Combination of Enzymes at the Optimum Conditions

The results obtained in the optimization of the US-assisted protocol allowed us to combine three different commercial enzymes under the optimal conditions (55 °C and pH 5.0 for 6 h) in order to prove their synergic efficiency to improve the total oil yield.

In other microalgae studies, authors have reported many results of enzymatic degradation without achieving very high cell disruption degrees, suggesting that for improving the enzymatic disruption of algal cells, the synergy of different kinds of processes and enzymes might be applied due to the rigid cell wall of these organisms [3,23,33,34,35].

**Figure 6:**
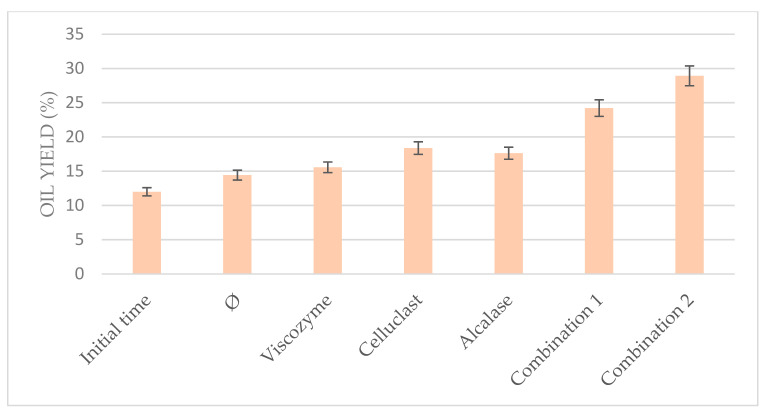
*Nannochloropsis* oil yield results using different combinations of enzymes with the US-assisted enzymatic pretreatment at the optimum conditions obtained, where Combination 1 corresponded to Celluclast+Viscozyme and Combination 2 corresponded to Celluclast + Viscozyme + Alcalase. Comparison with initial time and non-pretreated biomass (Ø) under the same conditions.

Indeed, when the enzymes were used separately in the present work, they achieve lower oil yield than when they were used in combination, combining either two or three of them, as can be seen in Figure 6. Celluclast in combination with Viscozyme achieved 24.22% oil yield, whereas the addition of Alcalase to this mixture achieved even higher results (28.92%), duplicating the results of the non-pretreated biomass and even triplicating those obtained in the initial time. This fact could be due to the *N. gaditana* cell composition in which proteins and biopolymers are binding; thus, the combination of Celluclast and Viscozyme commercial enzymes with a commercial protease as Alcalase could help to release the compounds of *N. gaditana* cells, facilitating the cell disruption. These results suggest that the combination of enzymes is a promising alternative for improving the enzymatic hydrolysis of *Nannochloropsis gaditana*.

### 3.6. Nannochloropsis gaditana Extracts Chemical Composition Analyzed by HPLC–ELSD

In order to study the lipid composition of the extracts, they were injected in an HPLC coupled with an ELSD detector resulting in analysis of different lipid classes. In the HPLC–ELSD chromatogram, seven peaks could be identified, which were attributed to nonpolar, neutral, and polar lipids. Peak 1 corresponds to triacylglycerides (TAG), diacylglycerides (DAG) for peak 2, free fatty acids (FFA) for peak 3, monoacylglycerols (MAG) for peak 4, glycolipids (GL) for peak 5, phosphatidylethanolamine (PE) for peak 6, and phosphatidylcholine (PC) for peak 7, which is in agreement with previous works on *N. gaditana* [3,10,34].

Lipid composition of the best extracts obtained by using US bath was thoroughly studied (Table 1) to compare lipid classes distribution and how it was affected by each pretreatment method showing clear differences between them. In general, it was observed an increase in the percentage of FFA and GL, which seemed to be logical due to an increased breakdown in the cell wall and the compounds bounded to it as polar lipids. Concretely, Viscozyme during 6 h in the US bath achieved a distribution highly different from the other conditions tested (*p* < 0.05), achieving a higher percentage of GL, PE, and FFA than the other extracts, which is in agreement with other works [21]. On the other hand, the rest of the extracts presented higher percentages of DAG, whereas their GL distribution was similar (*p* > 0.05), which suggest that using one pretreatment or another could highly change lipid classes distribution, obtaining a concentrated extract in one lipid class or another.

(%) Results were expressed as the individual relative percentage of each lipid species present in the sample (normalized areas).

**Figure 7:**
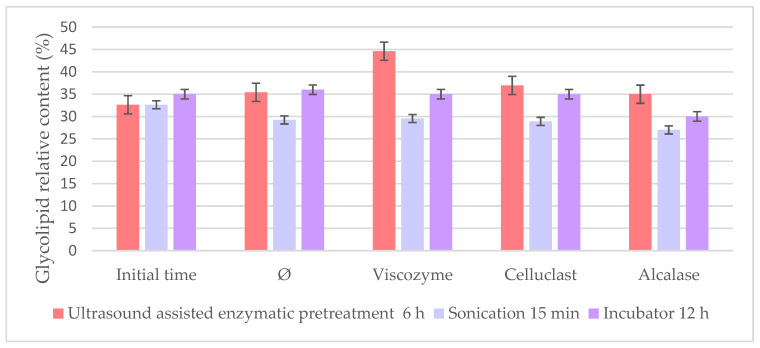
Glycolipid relative content (%) of *Nannochloropsis* extracts obtained using different pretreatment methods.

To further compare the different pretreatment conditions studied so far, the best methods based on the highest recovery oil yield were compared by their relative percentage of glycolipids since these polar lipids are of particular interest as lipids with interesting bioactivity. Extracts were obtained using the incubator at 12 h; the US bath at the best time (6 h) and high-intensity sonication at 15 min were compared, finding remarkable differences amongst enzymes and methods in the glycolipid content (Figure 7).

It is interesting to point out how the extracts with the highest oil yield obtained were less concentrated in glycolipid, as it can be seen in Figure 7, where all the enzymes achieved higher polar lipid content when the US bath was used. Polar lipids are usually bounded to the cell wall, which makes them more difficult to extract than other lipid classes, which could suggest that the enzymes extract them under less aggressive and longer conditions [6,36,37,38,39,40]. On the other hand, sonication as a more aggressive method is able to increase cell wall breakdown compared to other methods, being able to extract more lipid compounds together with GL, resulting in a lower GL concentration and a less selective extraction [23,41]. Consequently, it was shown the potential to combine the US bath with enzymatic pretreatment to enhance not only the total oil yield but also polar lipid content in the extracts and the usefulness of enzymes to produce extracts with novel composition [42], resulting in a fast and cost-effective combined method of pretreatment compared to conventional pretreatment methods.

Remarkably, Viscozyme achieved the highest glycolipid percentage (44.59%) in the US-assisted pretreatment, even though it was the one that achieved the lowest oil yield comparing the three enzymes in the US bath. This fact could be explained by Viscozyme being a commercial combination of different enzymes with high affinity to the different *Nannochloropsis* cell wall compounds, which has been described to be more useful than other enzymes alone breaking microalgal cells. Indeed, the cell wall in most of the green microalgae typically consists of polysaccharides (such as cellulose, pectin, and/or algaenan) and proteins (such as glycoproteins). Additionally, it has been shown in other works that multiple enzymes are needed to obtain the highest extraction efficiencies for other materials like *Nannochloropsis* sp. owing to its thick cell wall [23,24,27].

### 3.7. Scanning Electron Microscopy (SEM) Analysis of the Pretreated Biomass

**Figure 8:**
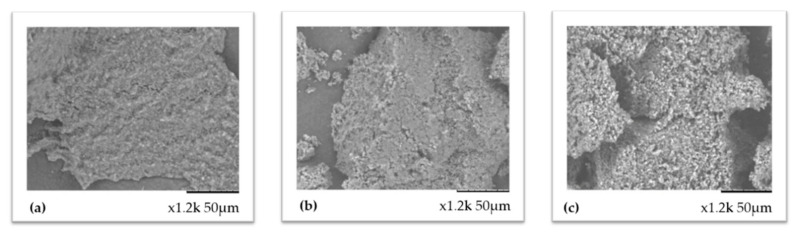
*Nannochloropsis* biomass analysis using SEM: (**a**) Initial time; (**b**) ultrasound pretreatment using US bath Elmasonic S 40H at 37 kHz for 6 h at 55 °C without enzymes; (**c**) combined US-assisted enzymatic pretreatment using US bath Elmasonic S 40H at 37 kHz for 6 h and 55 °C in combination with Alcalase, Viscozyme, and Celluclast commercial enzymes (together).

*Nannochloropsis* biomass after the enzymatic pretreatment was observed with a scanning electron microscope (SEM) to study morphological differences in the cell wall of the microalgae. Initial time biomass (non-pretreated biomass), pretreatment of 6 h in the US bath at 55 °C without enzymes, and the optimal US-assisted combined enzymatic pretreatment using Viscozyme, Celluclast, and Alcalase were prepared for analysis and compared. On the one hand, Figure 8a, which corresponds to the initial time, showed an intact cell wall material, whereas, in Figure 8b (pretreated biomass without enzymes), the material seemed slightly disturbed. The most surprising results were obtained with the combination of enzymes assisted by the US (Figure 8c), where the biomass seemed clearly damaged. In this case, the surface of the biomass was certainly different from the other pictures, showing a granular structure instead of a smooth structure, suggesting a higher disruption rate than in the other two samples. These pictures were in agreement with the produced oil yield, in which the results between the initial time and the pretreatment without enzymes hardly differed from each other, whereas the enzymatic pretreated sample almost triplicated the yield obtained in the non-pretreated biomass.

## 4. Application of the Pretreatment to Other Microalgae

**Figure 9:**
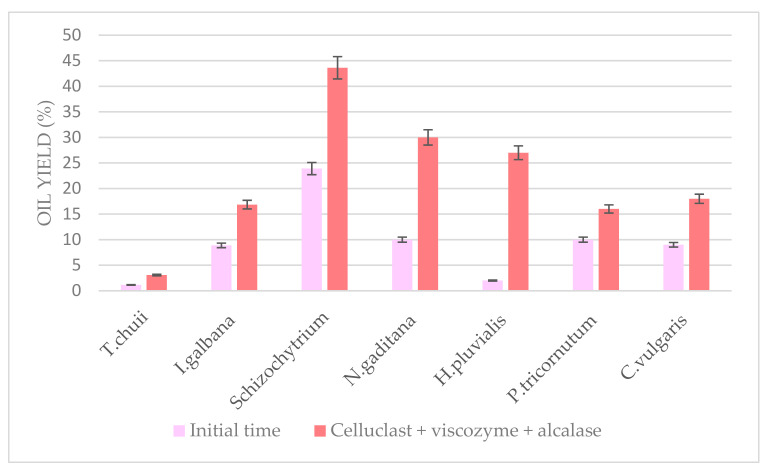
*Schizochytrium* spp., *I. galbana*, *T. chuii*, *C. vulgaris*, *H. pluvialis*, *P. tricornutum*, and *N. gaditana* biomass oil yield obtained at initial time and after the optimal US-assisted combined enzymatic pretreatment.

Optimum ultrasound-assisted combined enzymatic pretreatment at pH 5.0 and 55 °C for 6 h was applied to other microalgae biomass to study its effectivity. *Schizochytrium* spp., *Haematococcus pluvialis*, *Isochrysis galbana*, *Tetraselmis chuii*, *Chlorella vulgaris*, and *Phaeodactylum tricornutum* were compared to *N. gaditana* (Figure 9). Significant differences were observed at a 5% level between the different oil yields obtained from each biomass, which could be due to a highly diverse cell wall composition between each other. However, in general, for all the pretreated biomass, it could be observed how oil yield doubled comparing with their initial time. Taking into account the results shown, *Schizochytrium* would be the one with the less rigid cell wall as expected, which was easily broken, and the lipid fraction was easily extracted even at the initial time compared to the other microalgae.

These results show the high variation found between the microalgae species, which highlights the difficulty of using standard methods for all species and the importance of the development of a specific and effective pretreatment method for each one regarding cell wall composition and microalga behavior. This objective was achieved in this work for the microalga *N. gaditana*, although it could also be applied to similar algal biomass.

## 5. Conclusions

An effective ultrasound-assisted enzymatic pretreatment method for *N.gaditana* was developed, showing that efficacy of enzymes increased when they were combined at optimal conditions with a sonication method, instead of just incubating the enzymes with the microalgal biomass, and proving besides the synergistic effect of different types of enzymes in microalgal treatment. Optimum conditions for enzymes were chosen using 55 °C and pH 5.0 for 6 h in the US bath with different commercial enzymes. Combination of three hydrolytic enzymes at once under these pretreatment conditions rendered oil yields of almost 29%, notably improving the enzymatic hydrolysis of *Nannochloropsis gaditana* and suggesting a higher cell disruption rate, which was confirmed with SEM. In addition, optimized pretreatment and extraction process enhanced glycolipid distribution in *N. gaditana* extracts. Finally, the method was applied to other microalgal biomass of interest, such as *Schizochytrium* sp., *I. galbana*, and *T. chuii*. All the microalgae increased their oil yields by more than 100% when they were compared to the initial time, proving the applicability of the developed synergic process. Nonetheless, remarkable differences in the oil yield obtained for each biomass were also observed, though it was probably due to their different cell wall composition, showing the importance to adjust the pretreatment method separately for each different biomass, with different combinations of enzymes, at different experimental conditions (pH and temperature), and even in combination with physical methods.

## Figures and Tables

**Table 1:** Lipid classes distribution in different extracts obtained under traditional lipid extraction with the ultrasound-assisted enzymatic pretreatment at 6 h and analyzed by HPLC–ELSD, where triacyclglycerol (TAG), diacylglycerol (DAG), free fatty acid (FFA), glycolipid (GL), and phosphatidylethanolamine (PE) content are shown.

	TAG	DAG	FFA	GL	PE
Initial time	4.03 ± 0.46 ^a^	44.35 ± 0.82 ^a^	5.22 ± 0.33 ^a^	32.62 ± 0.87 ^a^	1.11 ± 0.08 ^a^
Ø	1.68 ± 0.87 ^a^	54.82 ± 0.97 ^b^	6.28 ± 0.27 ^a^	35.42 ± 0.59 ^a^^b^	1.79 ± 0.33 ^a^^b^
Viscozyme	2.13 ± 0.65 ^a^	38.96 ± 0.25 ^c^	12.03 ± 0.18 ^b^	44.59 ± 0.24 ^c^	2.90 ± 0.25 ^b^
Celluclast	2.38 ± 0.45 ^a^	49.19 ± 1.18 ^b^	8.94 ± 0.31 ^c^	36.96 ± 0.43 ^b^	2.53 ± 0.26 ^b^
Alcalase	3.69 ± 0.44 ^a^	48.68 ± 0.62 ^b^	9.28 ± 0.18 ^c^	34.99 ± 0.85 ^a^^b^	3.37 ± 0.23 ^b^
Combination 1	3.41 ± 0.39 ^a^	48.42 ± 0.55 ^b^	9.91 ± 0.32 ^c^	36.78 ± 0.41 ^b^	1.48 ± 0.18 ^a^
Combination 2	3.50 ± 0.51 ^a^	38.88 ± 0.48 ^c^	9.46 ± 0.27 ^c^	45.66 ± 0.36 ^c^	2.51 ± 0.22 ^b^

^a–c^ lowercase letters denote significant differences between each value in the same column (*p* < 0.05). Combination 1 corresponded to Celluclast+Viscozyme and Combination 2 corresponded to Celluclast + Viscozyme + Alcalase.
